# An Instrument for Measuring Social Participation to Examine Older Adults' Use of the Internet as a Social Platform: Development and Validation Study

**DOI:** 10.2196/23591

**Published:** 2021-05-17

**Authors:** Peter Anderberg, Linda Abrahamsson, Johan Sanmartin Berglund

**Affiliations:** 1 Department of Health Blekinge Institute of Technology Karlskrona Sweden; 2 Department of Health Sciences University of Skovde Skovde Sweden

**Keywords:** internet, older people, social participation, aging, instrument, elderly, social platform, perception, connectedness

## Abstract

**Background:**

Older people’s use of the internet is increasingly coming into focus with the demographic changes of a growing older population. Research reports several benefits of older people’s internet use and highlights problems such as various forms of inequality in use within the group. There is a need for consistent measurements to follow the development and use of the internet in this group and to be able to compare groups both within and between countries, as well as follow the changes over time.

**Objective:**

The aim of this study was to create an instrument to measure an older person’s perception of the benefits of their online social participation, unconnected to specific applications and services. The instrument to measure internet social participation proposed in this paper builds on social participation factors and is a multidimensional construct incorporating both social relations and societal connectedness.

**Methods:**

A short instrument for measuring social participation over the internet was created. An exploratory factor analysis (EFA) was conducted in a random selection of persons aged 65 years or older (n=193) on 10 initial items. Further validation was made by confirmatory factor analysis (CFA) in the remaining group (n=193).

**Results:**

A 1-factor solution for the social internet score was decided upon after exploratory factor analysis (EFA; based on a random sample of half the data set). None of the questionnaire items were excluded based on the EFA, as they all had high loadings, the lowest being 0.61. The Cronbach α coefficient was .92. The 1-factor solution explained 55% of the variance. CFA was performed and included all 10 questionnaire items in a 1-factor solution. Indices of goodness of fit of the model showed room for improvement. Removal of 4 questions in a stepwise procedure resulted in a 6-item model (χ^2^_6_=13.985; χ^2^/degrees of freedom=1.554; comparative fit index=0.992; root mean square error of approximation=0.054; standardized root mean square residual=0.025).

**Conclusions:**

The proposed instrument can be used to measure digital social participation and coherence with society. The factor analysis is based on a sufficient sample of the general population of older adults in Sweden, and overall the instrument performed as expected.

## Introduction

Older adults’ internet use is the focus of an increasing number of research studies [1]. Internet use has been reported to promote the well-being [2] and active aging [3] among older people and to act as a possible support for maintaining cognitive function [4,5]. Despite these benefits, there are also reports of a lower percentage of internet users in older age groups than in the whole population [6,7], creating a digital divide, leaving out many older adults from the benefits of the online world.

Research on this digital divide has initially been focused on actual internet access (first-level digital divide) and internet skills and use (second-level digital divide) [8]. However, with an increasing number of older people getting access to the internet, the focus has also shifted to a third-level digital divide in which the tangible outcomes of internet use are highlighted [8] and where actual users also differ in how they benefit from their online presence. One such outcome is the use of the internet to maintain social contacts and avoid loneliness [9-11].

The positive relationship between social participation and well-being and health is well documented [12,13]. Social participation has no universally agreed definition but is generally measured in terms of the quantity or quality of social interactions and connections [14]. In a study on older adults’ social participation, Utz et al [15] classified it in the realm of organizational affiliations, friendship ties, kinship networks, social connectedness, social support, or social integration. Internet use has been generally acknowledged to have the potential to support such social affordance [16-19].

A possibility to measure the online social participation, unconnected to specific applications and services, would be of interest for comparing groups both within and between countries, and to examining changes over time. The instrument to measure internet social participation proposed in this paper builds on social participation factors and is a multidimensional construct incorporating both social relations and societal connectedness. It focuses on the subjective feeling of social participation free from references to specific applications and services.

## Methods

### Data Collection and Sample

Data were obtained from a sample of participants in the Swedish National Study of Aging and Care (SNAC). SNAC is a longitudinal cohort study of a representative sample of the aging Swedish population that started its data collection in 2001. It is a comprehensive, interdisciplinary study that investigates the health and living conditions of the Swedish population aged 60 years and older. A detailed outline of the SNAC study is available from Lagergren et al [20]. The present study sample is based on participants from 1 of the 4 regions in the SNAC study, the SNAC Blekinge (SNAC-B) cohort with individuals living in the municipality of Karlskrona.

As an addition to this study, it was seen necessary to further the efforts to gather information about older persons' use and experiences of societal digitalization. A first questionnaire was sent out in 2017, and the second slightly modified questionnaire (the base for this study) was sent out in April 2019 to all participants in the SNAC-B study (N=733). A total of 581 persons responded, corresponding to a response rate of 79.3% (581/733). In the present study, only individuals who responded that they were information and communication technology users were included (n=393).

Out of the internet users, 388 were responders of at least one social participation score item. Of these, 21 persons had missing answers on at least one of the 10 questionnaire items in the social participation score (15 persons had 1 missing question; 3 persons had 2 missing; and 1 person each had 5, 7, and 9 missing). From the answers of the 388 responders, the following were the number of missing values for the 10 questions: 4, 3, 3, 8, 5, 5, 3, 3, 5, and 3, respectively. Finally, persons answering fewer than 5 questions were excluded, rendering the final number of participants to 386.

### Instrument Development

In the first questionnaire sent out in 2017, there was a focus on older persons' affinity to technology, so-called technophilia [21], and this research was subsequently reported in JMIR. In further analysis of this initial research, the need was also found for an instrument that could measure experiences of the online social participation, unconnected to specific applications and services. This would be of interest to see changes over time between our cohorts (eg, asking about a specific application as Facebook use today could be changed to another application over time, but the underlying latent construct is the same). Social participation [14,15] was chosen as a theoretical framework incorporating a multidimensional construct with both social relations and societal connectedness.

A list of possibly interesting and relevant aspects of social participation and connectedness in an online environment was constructed from the theoretical framework and the control questions in the questionnaire (Table 1). From this, an initial 10-question instrument (5 questions in each domain) was composed by PA and JSB, who have expertise in the field of aging and the internet. This list was reviewed together with a third expert, in the psychology of aging, and different aspects and formulations were discussed and agreed upon.

The questionnaire was then pretested for face validity, coherence, and understandability with cognitive interviews [22] being conducted with the target group (a convenience sample of 6 individuals of both sexes ranging in age from 66 to 80 years). The interviewees were given the 10-item questionnaire and were encouraged to think aloud when they read the questions. The interviewer would also follow up with verbal probing (ie, questions about how well the interviewee understood the question) based on item wording, terminology, and if the structure was clear and easy to understand. Specifically, the questions “Can you repeat the question I just asked in your own words?”, “Was there anything confusing about this question?”, “What does the word [term] mean to you as it is used in the question?”, and “Tell me what you thought when I asked about [topic of question],” were asked.

The item questions were then revised according to the feedback from the interviews with respect to the verbal probing. Especially important was to make sure that the questionnaire was using terminology relevant to older people using technology to ensure face validity. The questionnaire was translated into English using backward-forward translation from the original Swedish version, by native-speaking translators in each direction.

The resulting questionnaire items can be found in Table 1.

**Table 1:** Initially suggested 10-question instrument.

Domain	English (translation)	Swedish (original)
Social relations	1. I think the internet helps me stay in touch with friends and family	1. Jag tycker att internet hjälper mig att hålla kontakt med vänner och familj
Societal connectedness	2. I think the internet helps me keep up to date with what's happening in society	2. Jag tycker att internet hjälper mig att hålla mig informerad om vad som händer i samhället
*Societal connectedness* ^a^	*3. I think the internet makes me feel more included in society*	*3. Jag tycker att internet gör att jag känner mig mer delaktig i samhället*
Social relations	4. I think the internet makes me feel less lonely	4. Jag tycker att internet gör att jag känner mig mindre ensam
*Societal connectedness*	*5. I think the internet helps me to access meaningful activities*	*5. Jag tycker att internet hjälper mig att få tillgång till meningsfulla aktiviteter*
*Social relations*	*6. I think the internet helps me keep up my social network*	*6. Jag tycker att internet hjälper mig att hålla igång mitt sociala nätverk*
*Societal connectedness*	*7. I think the internet helps me reconnect to old memories and events of yesteryear*	*7. Jag tycker att internet hjälper mig att återknyta till gamla minnen och händelser från förr*
Societal connectedness	8. I think the internet helps me find pastimes and amusement	8. Jag tycker att internet hjälper mig att hitta nöje och förströelse
*Social relations*	*9. I think the internet helps me to expand and create new social networks*	*9. Jag tycker att internet hjälper mig att utöka och skapa nya sociala nätverk*
*Social relations*	*10. I think the internet makes me feel less isolated*	*10. Jag tycker att internet gör att jag känner mig mindre isolerad*

^a^The questions that made it into the final instrument are indicated by italics.

### Statistical Analysis

In order to be able to include all participants who answered at least five questions on the social participation score, imputation was done on the missing values for the 10 items to be used in creating the social participation score. Thus, 26 missing values were imputed by using median imputation across the individuals. As the missingness was limited (close to 0.5%) it is not likely that the choice of imputation method would affect statistical analyses to any noticeable extent. To compare, a complete case analysis would result in excluding 5% of the 386 participants and was not preferable.

After imputation was carried out, a random sample (n=193) was drawn to perform an exploratory factor analysis (EFA), with the main aim of deciding on a single- or multiple-factor solution, and of delimiting the number of items making up the social participation score. The number of factors to include were decided based on the Cattell scree test [23] and Horn parallel analysis [24], along with inspection of the factors. After the EFA had been carried out, the Cronbach α coefficient was calculated for examining the internal consistency of the questionnaire items within a factor [25]. On the other half of the data (n=193), a confirmatory factor analysis (CFA) was performed, based on the structure proposed by the EFA. As the CFA was carried out on another data sample, it was possible to cross-validate the measurement model proposed by the EFA, as done by Kamin and Lang [26]. Before any of the factor analyses were carried out, both the Kaiser-Meyer-Olkin measure of sampling adequacy, which calculated the proportion of the variance in the questionnaire items that might be caused by underlying factors [27], and the Bartlett test of sphericity, which tested the hypothesis that the items are unrelated [28], were performed. These tests were conducted on the complete data set (N=386).

Regarding sample sizes, Floyd and Widaman [29] recommend a sample size of at least 10 participants per parameter estimated (here meaning the number of questions in the questionnaire) for performing a CFA. The same recommendation is made by Nunnally [30] for the EFA. In the CFA, several goodness-of-fit measures were assessed: the chi-square statistic by itself and relative to the degrees of freedom (ideally <2 [31]), the standardized root mean square residual (SRMR; ideally <0.05 [31]), the comparative fit index (CFI; acceptable fit >0.95, [31]), and the root mean square error of approximation (RMSEA; ideally <0.05; acceptable fit 0.05-0.08, [32]). Modification indices (MIs) were evaluated to possibly improve the model by limiting the number of questionnaire items and to identify complex items in a similar manner to Hyde et al [33]. MIs are values corresponding to univariate score tests and reflect the model fit improvement that would be present if a constrained parameter were to be estimated as a free one [34]. In particular, we looked at MIs for covariances between questionnaire items within a factor in CFAs and hypothesized that for significant score tests, instead of allowing for covariances in the model between items, we could exclude 1 of 2 highly correlated items.

A total score, with continuous values from 1 to 5, of a 1-factor solution, was calculated with the nonrefined method of making a weighted sum score [35], with weights decided by the standardized factor loadings from the CFA. We decided not to use refined methods, such as regression scores or Bartlett scores, as a nonrefined method is more stable across samples [36]. The total score was used to delineate 5 different categories of 0.8 units each as the dependent variable in univariate ordinal logistic regression models [37]. This was done for evaluating associations between the social participation score and independent variables, such as gender and internet use frequency. Note that in such models, the magnitude of the β coefficients cannot be interpreted. Correlations between the social participation score and 3 other technical scores, TechPH (“an instrument for measuring older people's attitudes towards technology” [21]) and eHEALS (“measuring consumers’ combined knowledge, comfort, and perceived skills at finding, evaluating, and applying electronic health information to health problems” [38]), were also assessed, along with approximate *P* values. The first of the scores, consisting of two factors, was previously developed within our research group. Further validation of the score was made by evaluating correlations between the score (and the individual questions within the score) and other variables meant to measure neighboring (eg, usage of social networking sites) as well as diverse (eg searching of health-related information) quantities.

All statistical analyses were performed in SPSS (IBM Corporation) or R (R Foundation for Statistical Computing) software, using packages Psych, lavaan, REdaS, MASS, and Hmisc.

## Results

### Exploratory Factor Analysis

The Kaiser-Meyer-Olkin measure of sampling adequacy was 0.91 for the complete data, and Bartlett test of sphericity was highly significant (*P*<.001). Thus it was appropriate to perform factor analyses.

A 1-factor solution for the social internet score was decided upon after the EFA (based on a random sample of half the data set). This was suggested by the bend in the scree plot, and also, only the first factor had an eigenvalue >1, although 3 factors were proposed based on the parallel analysis. However, it can be noted that the result from the parallel analysis was on the border of proposing a 1-factor solution: the eigenvalue for the second factor in the actual data was not much higher than that for the simulated or resampled data. When we inspected the factor solutions that had more than one factor, many items were cross-loaded on several factors, making the solutions difficult to interpret (Table 2).

**Table 2:** Two-factor solution with (standardized) cross-loadings from exploratory factor analysis on the sampled group (n=193).

Question	Factor 1 loadings	Factor 2 loadings
1. I think the internet helps me stay in touch with friends and family	0.36	0.66
2. I think the internet helps me keep up to date with what's happening in society	0.22	0.88
*3. I think the internet makes me feel more included in society* ^a^	0.52	0.66
4. I think the internet makes me feel less lonely	0.68	0.35
*5. I think the internet helps me to access meaningful activities*	0.53	0.41
*6. I think the internet helps me keep up my social network*	0.7	0.42
*7. I think the internet helps me reconnect to old memories and events of yesteryear*	0.72	0.31
8. I think the internet helps me find pastimes and amusement	0.45	0.56
*9. I think the internet helps me to expand and create new social networks*	0.71	0.31
*10. I think the internet makes me feel less isolated*	0.84	0.26

^a^The questions that made it into the final instrument are indicated by italics.

The number of cross-loadings depends on which cutoff value to choose for item loadings to be kept within a factor. There is no general rule of thumb regarding this value, rather several different rules exist and have been reported in the literature [26,39,40]. To retrieve 2 factors without cross-loadings, a cutoff value of >0.52 would give such a solution. It can, however, be noted that Questions 3, 5, and 8 have similar loadings on both factors. Also, it is difficult to understand what the difference in theoretical construct between the 2 factors would be, given their included questionnaire items. Based on this, a 1-factor solution was decided upon. None of the questionnaire items were excluded based on the EFA (and the cutoff value for factor loadings was 0.5, the lowest value recommended by Hair et al [40]) as they all had high loadings, the lowest being 0.61 (Table 3). The Cronbach α coefficient was .92, and the 1-factor solution explained 55% of the variance.

### Confirmatory Factor Analysis

A CFA was performed that included all 10 questionnaire items in a 1-factor solution (based on the second, nonsampled part of the data); the standardized factor loadings can be seen in Table 3). Results indicated that there is room for improvement based on the indices showing the goodness of fit of the model. After inspection of the model’s MI values, it was found that some items had high correlations which could cause some of the model misspecifications. As the MI values represent score test statistics, by following a chi-square distribution of 1 degree of freedom, the values should be kept small if no model misspecification were present. We used a stepwise procedure in which we removed 1 of the questionnaire items with the highest MI value (>10). When 1 item was removed, a new CFA was performed on which the new MI values were calculated. The problematic items were Questions 4 and 10 (MI=36.349), Questions 2 and 3 (MI=35.049), Questions 5 and 8 (MI=19.049), and Questions 1 and 6 (MI=11.589). Removing the questionnaire items with the lowest standardized factor loadings led to the exclusion of Questions 4, 2, 8, and 1. The removal of questions was agreed upon by medical and technical experts in the field. The standardized factor loadings in the CFA based on the 6 remaining questionnaire items are shown in Table 3.

Note that a parallel analysis (EFA) proposed a 1-factor solution for the 6 questionnaire items being left out of the 10 original ones.

**Table 3:** Standardized factor loadings from exploratory and confirmatory factor analyses for the 1-factor solution based on the original 10 questionnaire items and the chosen 6 items after exclusions. Based on the 2 different parts of the data: sampled (EFA) and nonsampled (CFA) groups.

Question	EFA^a^ (n=193)	CFA^b^ (n=193)	CFA with 6 items (n=193)
1. I think the internet helps me stay in touch with friends and family	0.69	0.58	—^c^
2. I think the internet helps me keep up to date with what's happening in society	0.69	0.59	—
*3. I think the internet makes me feel more included in society* ^d^	0.82	0.73	0.69
4. I think the internet makes me feel less lonely	0.75	0.75	—
*5. I think the internet helps me to access meaningful activities*	0.68	0.78	0.75
*6. I think the internet helps me keep up my social network*	0.81	0.80	0.84
*7. I think the internet helps me reconnect to old memories and events of yesteryear*	0.75	0.80	0.80
8. I think the internet helps me find pastimes and amusement	0.71	0.72	—
*9. I think the internet helps me to expand and create new social networks*	0.75	0.80	0.84
*10. I think the internet makes me feel less isolated*	0.80	0.77	0.75

^a^EFA: exploratory factor analysis.

^b^CFA: confirmatory factor analysis.

^c^Not applicable.

^d^The questions that made it into the final instrument are indicated by italics.

Indices of goodness of fit of the model were improved for the 6-item model compared to the 10-item model. Following are the indices for the 6-item model: χ^2^_6_=13.985; χ^2^/degrees of freedom=1.554; CFI=0.992; RMSEA of approximation=0.054; SRMR=0.025).

The distribution of the social participation score among the 386 persons in the study is presented as means and fractions of participants receiving scores in 5 different ordered categories from 1 to 5, representing rounded score values of 1-1.8, 1.9-2.6, 2.7-3.4, 3.5-4.2, and 4.3-5, respectively (Table 4). Beta coefficients and *P* values from univariate ordinal logistic regression models, in which the categories 1-5 were used for the social participation score, are presented in Table 4.

Study participants with a medium level of education (finished secondary school, but without higher education except vocational training) had significantly lower social participation scores than did the participants with a low level of education (did not finish secondary school). A higher social participation score was also associated with more frequent use of the internet, as well as higher use of internet services.

Pearson correlation coefficients and approximate *P* values that compared the social participation score to the TechPH scores were divided into the factors TechEnthusiasm, TechAnxiety [21], and eHEALS [38], and were 0.480 (*P*<.001) for TechEnthusiasm; –0.159 (*P*=.003) for TechAnxiety, and 0.372 (*P*<.001) for eHEALS. Correlations comparing the social participation score (and its individual question items) to variables measuring a variety of internet-related quantities are presented in Table 5. The score is correlated to variables measuring, for example, whether the participant has been listening to web radio, participating on social networking sites, or following news reporting online. The score showed a lower or no significant correlation with health-related measures and no correlation with participation in online auctions or usage of Mobile BankID (Mobile BankID is an electronic identification solution in Sweden that allows companies, banks, organizations, and governments agencies to authenticate and conclude agreements with individuals over the internet).

**Table 4:** Distribution of social score among the study participants and associations between social score and independent variables presented as β coefficients from univariate ordinal logistic regression models.

Variable		Distribution, n (mean)	Fractions in categories 1-5	β value in ordinal logistic regression	*P* value
All		386 (3.05)	16, 21, 25, 23, 16	—^a^	—
**Gender**					
	Men	186 (3.09)	17, 19, 23, 23, 19	Ref cat^b^	—
	Women	200 (3.01)	15, 22, 27, 24, 13	−.15	.40
**Age**					
	<75 years	195 (3.09)	13, 21, 28, 23, 15	Ref cat	—
	≥75 years	191 (3.00)	19, 20, 22, 23, 16	−.14	.44
**Education^c^**					
	Low	103 (3.24)	11, 22, 22, 20, 24	Ref cat	—
	Medium	139 (2.88)	21, 19, 27, 23, 10	−.57	.01
	High	124 (3.04)	15, 22, 23, 24, 15	−.29	.20
**Internet use frequency**					
	Low	44 (2.3)	39, 30, 11, 14, 7	Ref cat	—
	High	342 (3.14)	13, 20, 27, 24, 17	1.38	<.001
**Use of internet services**					
	Low	55 (2.36)	42, 22, 15, 16, 5	Ref cat	—
	Medium	240 (2.98)	14, 24, 28, 23, 12	1.15	<.001
	High	91 (3.64)	5, 11, 23, 29, 32	2.27	<.001

^a^Not applicable.

^b^Ref cat: reference category.

^c^Education was categorized in three groups according to the previous Swedish education system, relevant for the age groups in this study: low, those who did not finish secondary school; medium, those who finished secondary school but no further education; high, those with some form of higher education.

**Table 5:** Pearson correlations between the social participation score (and its individual question items) to variables measuring a variety of internet-related quantities.

Item	Social sites^a^, *r*	News and Media, *r*		Information^d^, *r*	Activities^e^, *r*	Economic utilities, *r*			Health utilities, *r*	
		Web radio^b^	News sites^c^			Mobile BankID, and other ID^f^	Buying and selling^g^	Web search^h^		EMR^i^
I think the internet makes me feel more included in society	0.291**	0.258**	0.313**	0.193**	0.179**	0.111*	0.046	0.169**		0.039
I think the internet helps me to access meaningful activities	0.189**	0.207**	0.152**	0.160**	0.168**	0.093	0.080	0.138**		0.104*
I think the internet helps me keep up my social network	0.366**	0.209**	0.207**	0.196**	0.109*	0.107*	0.031	0.090		0.102*
I think the internet helps me reconnect to old memories and events of yesteryear	0.264**	0.164**	0.204**	0.151**	0.157**	−0.029	0.038	0.095		0.067
I think the internet helps me to expand and create new social networks	0.401**	0.224**	0.199**	0.102*	0.166**	0.086	0.069	0.074		0.058
I think the internet makes me feel less isolated	0.266**	0.248**	0.230**	0.103*	0.104*	0.057	0.049	0.135**		0.110*
Internet social participation score	0.364**	0.264**	0.262**	0.182**	0.178**	0.084	0.063	0.140**		0.098

*Correlation is significant at *P*<.05 (2-tailed).

**Correlation is significant at *P*<.01 (2-tailed).

^a^Full question: Have you participated in social networking sites such as Facebook or Twitter and created a user profile and made posts or chatted?

^b^Full question: Have you been listening to web radio?

^c^Full question: Have you been looking at news sites?

^d^Full question: Have you been looking for information about products or services?

^e^Full question: Have you been playing or downloading games, pictures, movies or music?

^f^Full question: Have you been using Mobile BankID or any other electronic identification?

^g^Full question: Have you been selling goods or services through net auction sites like eBay?

^h^Full question: Have you been searching for information on diseases or treatments on official or private websites?

^i^Full question: Have you been looking at your electronic medical records (EMR) via an internet portal?

## Discussion

### Principal Results

We chose a 1D final factor structure for the social participation as supported by the scree plot but not entirely by the parallel analysis. However, in order to make the score user friendly, it was important to exclude factors with many cross-loadings, which otherwise would have led to difficulties in interpreting the underlying constructs. However, the 1D structure for the 6 final questionnaire items was supported by both the scree plot and the parallel analysis.

In removing the 4 questions (1, 2, 4, and 8), the loss of information was thought to be limited. By inspection of the questions, clear similarities can be seen in Questions 4 and 10 (feeling of loneliness and feeling of isolation), Questions 2 and 3 (being informed about society and taking part of society), Questions 5 and 8 (access to meaningful activities and finding pleasure and amusement), and Questions 1 and 6 (keeping contact with friends and family and keeping up my social network). The goodness-of-fit indices were clearly improved after the simplification of questionnaire items.

The final instrument was shortened to 6 items in a 1-factor score, making it easy to use for any survey that measures digital social participation. It builds on social participation factors and has a multidimensional construct, incorporating both social relations and societal connectedness. Also, it focuses on the subjective feeling of social participation. This universal approach, unconnected to specific applications or services, suggests that its use will be able to compare different groups and examine changes over time.

The proposed digital social participation score showed no significant association with gender or age among older adults, thus demonstrating that it has the capacity to serve as a general instrument. It complies well with assumptions about internet use: a higher social participation score is associated with more frequent use of the internet and higher use of internet services.

In different domains, the score correlates well with social internet activities and activities that promote social coherence and correlates poorly with more instrumental activities (eg, following news reports online or participating on social networking sites vs participating in online auctions or using Mobile BankID).

To conclude, we believe the proposed instrument can be used to measure digital social participation and coherence with society. The factor analysis is based on a sufficient sample of the general population of older adults in Sweden, and overall, the instrument performed as expected.

Whether it can be used to detect differences in outcomes such as loneliness, depression, or sense of coherence [41] needs to be shown in further studies. The instrument must also be validated in different contexts, such as in other populations and countries.

### Limitations

Performing the final CFA on the data set that was used to redefine the theoretical model by the modification indices limited the validity of the goodness-of-fit measures. The social participation score should ideally be confirmed on another data set in a future study.

## Abbreviations

CFI: comparative fit index
EFA: exploratory factor analysis
MI: modification index
RMSEA: root mean square error
SNAC: Swedish National Study of Aging and Care
SNAC-B: Swedish National Study of Aging and Care Blekinge
SRMR: standardized root mean square residual
